# Recent Progress in Understanding the Mechanism of Formate Dehydrogenases

**DOI:** 10.1002/cbic.70555

**Published:** 2026-10-06

**Authors:** Russ Hille, Dimitri Niks

**Affiliations:** ^1^ Department of Biochemistry University of California Riverside California USA

**Keywords:** formate dehydrogenase, hydride transfer, molybdenum, reaction mechanism

## Abstract

Recent work concerning the reaction mechanism of the molybdenum‐containing formate dehydrogenases is discussed, focusing on two alternate mechanisms of action for the enzyme involving either formation of CO_2_ or bicarbonate as the immediate product of the reaction. On the basis of the pH dependence of the rate of reduction of enzyme by formate and the results of a recent GC/MS study undertaken under single‐turnover conditions indicating that no ^18^O from solvent H_2_^18^O is incorporated into product CO_2_, it is concluded that bicarbonate cannot be formed in the course of the reaction, and that cysteine, once dissociated from the molybdenum, cannot be functioning as a base in the course of the reaction.

## Graphical abstract

A hydride transfer mechanism for the molybdenum‐ and tungsten‐containing formate dehydrogenases.

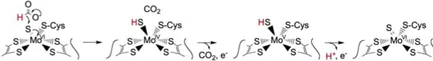

1

The molybdenum‐ and tungsten‐containing formate dehydrogenases have drawn considerable attention due to their ability to catalyze the reversible interconversion of formate and CO_2_, either generating reducing equivalents in the forward direction or consuming them in the reverse [1, 2]. Understanding the manner in which these enzymes catalyze this interconversion at room temperature and atmospheric pressure is likely to provide design principles guiding the development of a new generation of synthetic catalysts that will be critical in the new energy economy.

The cytosolic FdsDABG formate dehydrogenase from *Cupriavidus necator* and other organisms has the structure shown in Figure 1 [1, 3]. The active site molybdenum center lies in the C‐terminal domain of the FdsA subunit and has an L_2_MoS(S‐Cys) active site in a trigonal prismatic coordination geometry. The N‐terminal portion of FdsA has four [4Fe‐4S] clusters and one [2Fe‐2S] cluster. FdsB has a [4Fe‐4S] cluster and FMN (the site of NAD^+^ reduction to NADH) and FdsG has a final [2Fe‐2S] cluster. The cofactorless FdsD appears to stabilize the FdsA domain containing the molybdenum center.

**FIGURE 1:**
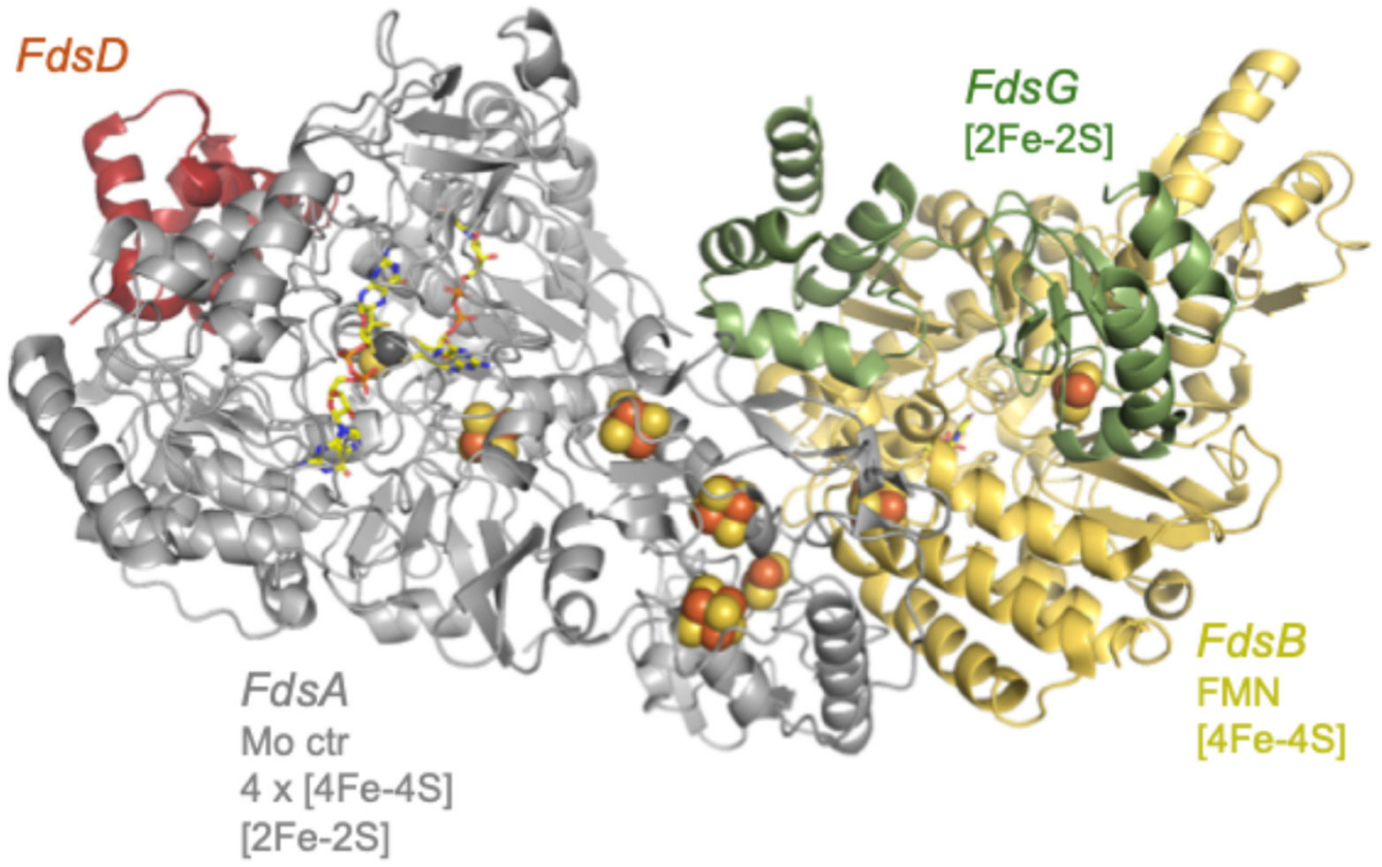
The structure of the cytosolic FdsDABG formate dehydrogenase (PDB 1FDO). Shown is the cryoEM structure of the enzyme from *Rhodobacter capsulatus* [3], which largely confirms the model previously proposed for the *C. necator* enzyme on the basis of homologies to the *Thermus thermophilus* NADH dehydrogenase [1].

Several reaction mechanisms for the molybdenum‐ and tungsten‐containing formate dehydrogenases, with either cysteine or selenocysteine coordinated to the metal, have been proposed over the years (for reviews, see [4, 5]). For example, Khangulov et al. [6] have proposed that an active site histidine deprotonates the Cα of formate to bring about reduction of the molybdenum center. Mota et al. [7] have suggested a sulfur shift mechanism in which the terminal Mo═S is transferred to the (seleno)cysteine to give a persulfide, with the system passing through a thiocarbamate intermediate in the process of reducing the molybdenum center. Raaijmakers and Romao [8] have proposed that formate displaces the (seleno)cysteine coordinating the molybdenum, which then abstracts the Cα proton of substrate to bring about reduction of the molybdenum center. Leimkühler and coworkers [9] have similarly proposed a mechanism in which formate displaces the cysteine from the molybdenum coordination sphere, which then abstracts the Cα proton to initiate reduction of the molybdenum center. Finally, using QM/MM calculations, Dong and Ryde [10] have suggested that formate does not bind directly to the molybdenum but is oxidized by formation of a thiocarbamate at the (seleno)cysteine, transferring a hydride to the Mo═S moiety in the process to reduce the molybdenum center.

In 2016, Niks et al. [11] proposed a simple hydride‐transfer reaction that has become widely (if not universally) accepted. As shown in Figure 2A (top), the reaction proceeds by direct hydride transfer from the Cα of formate to the Mo═S to bring about the reduction of the molybdenum center and direct formation of CO_2_. The mechanism avoids the need to deprotonate the formate Cα (formamide has a pK_a_ of 34, and formate would be expected to have an even higher one) and readily accounts for the observation that the substrate hydrogen is transferred to a site that is strongly magnetically coupled to the molybdenum in the Mo(V) state. Recently, however, an alternative mechanism has been proposed (Figure 2B, bottom) in which the active site cysteine is displaced by hydroxide from solvent, which then functions as a base to facilitate nucleophilic attack of the Mo–OH on the carbonyl group of formate to yield bicarbonate, which then dehydrates to the observed CO_2_ product [12]. To account for the observation that no ^18^O was observed to be incorporated into product CO_2_ when the reaction was carried out in H_2_^18^O under multiturnover conditions, it was proposed that a sequestered water molecule in the active site of the enzyme was recycled with each catalytic turnover and did not exchange with solvent.

**FIGURE 2:**
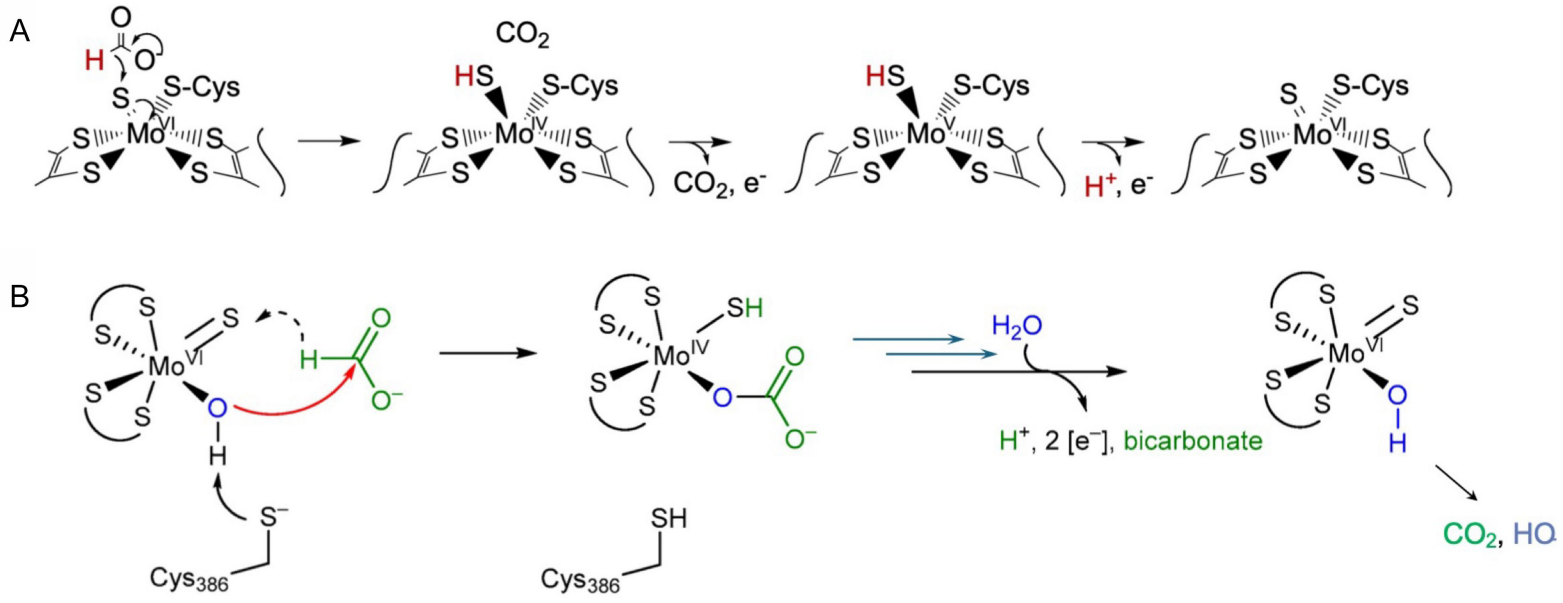
Alternate mechanisms for the reaction catalyzed by formate dehydrogenase. Shown are the alternate mechanisms proposed by Niks et al. [11] (A, top) and Kumar et al. [12] (B, bottom).

In order to assess whether such a sequestered water molecule might exist, a GC/MS study has been undertaken under single‐turnover conditions after overnight incubation of the enzyme in 70% enriched H_2_^18^O, reacting the enzyme with one equivalent of ^13^C,^2^H‐formate on ice (to slow the reaction) [13]. The theoretical expectation for enzyme‐catalyzed ^18^O incorporation into product CO_2_ from solvent under these conditions is 47% (2/3 of 70% given that, assuming bicarbonate is formed, there is a one in three chance that the newly incorporated ^18^O will be lost on dehydration to CO_2_). At 30 s, only 16% ^18^O incorporation occurred, increasing to ~39% after 70 s. Since the enzymatic reaction is expected to be complete within 200 ms, the significant extent of observed incorporation of ^18^O into CO_2_ is mostly if not entirely postcatalytic, occurring subsequent to the enzyme‐catalyzed generation of CO_2_. This conclusion is consistent with the earlier work under turnover conditions, where the observed incorporation of ^18^O was far less than expected [12]. Although this postcatalytic exchange was considerably faster than the known exchange rate at pH 7.5 on ice with a half‐time >5 min (estimated from Johnson et al. [14] and Ho and Sturtevant [15]), it is clear that on the timescale of the experiment any label introduced by the enzyme would not exchange back out. In control experiments at either 30 s or 70 s, little noncatalytic exchange of ^18^O from solvent into ^13^CO_2_ was found [13]. Somewhat surprisingly, the observed extent of postcatalytic ^18^O exchange into CO_2_ from solvent was largely unchanged when the reaction was performed with enzyme that had been inactivated with cyanide. This work demonstrates clearly that bicarbonate is not the immediate product of formate oxidation by FdsDABG (and presumably other molybdenum‐ and tungsten‐containing formate dehydrogenases, whether they contain cysteine or selenocysteine), a conclusion originally arrived at by Khangulov et al. [6] and also supported by the more recent electrochemical work of Meneghello et al. [16, 17].

In addition to the above GC/MS work, the pH dependence of the reductive half‐reaction of the FdsDABG formate dehydrogenase is inconsistent with a mechanism involving base‐catalysis, as depicted in Figure 2B (bottom). The reaction of anaerobic, oxidized FdsDABG with formate exhibits multiple phases, the fastest of which reflects reaction of fully oxidized enzyme with the first equivalent of formate, exhibiting a hyperbolic dependence on [formate] throughout the pH range investigated. Figure 3 shows a plot of the limiting *k*_fast_ as a function of pH and clearly indicates a (rather weak) acid‐catalyzed rather than base‐catalyzed reaction, with *k*_fast_ decreasing from ~500 to ~200 s^−1^ in going from pH 6 to 9 [13].

**FIGURE 3:**
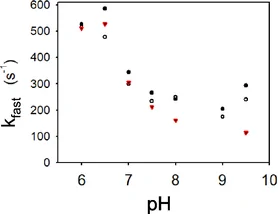
The pH dependence of the fast phase of FdsDABG reduction by formate. Shown is a plot of the limiting *k*_fast_ for the fast phase of the reaction at high formate concentrations versus pH [13].

Most recently [18], the FdsDABG formate dehydrogenase has been reexamined by X‐ray absorption spectroscopy using enzyme that was ~95% functional, past work [19] having suffered from the low specific activity of the enzyme used, with spurious Mo═O groups inserted into the molybdenum coordination sphere of the inactive, presumably desulfo form of the enzyme. The more recent XAS work also employed the high‐energy resolution fluorescence detected technique. Surprisingly, no evidence for a short Mo═S bond is seen in the EXAFS of oxidized enzyme, which was best fit with five Mo─S at a distance of 2.375 Å and a sixth at 2.557 Å (along with four Mo─C at 3.364 Å due to the enedithiolate carbons). On reduction, the longer Mo─S at 2.557 Å was lost and replaced by a new shorter Mo─S at 2.528 Å. Significantly, the molybdenum X‐ray K‐edge did not shift perceptibly on reduction of the oxidized enzyme by formate, as expected for a shift from Mo(VI) to Mo(IV). The data were interpreted as indicating that the cyanolyzable sulfur of oxidized enzyme was present not as a Mo^VI^═S but as the terminal sulfur of a cysteine persulfide coordinated in a side‐on ε_2_ fashion in an L_2_Mo^IV^(S‐SCys) configuration. Reduction by formate results not in further reduction of the metal but splitting of the persulfide bond to yield the canonical L_2_Mo^IV^(SH)(S‐Cys) configuration for reduced enzyme, the point being that the oxidation‐reduction chemistry is happening in the ligand field of the metal, not the metal itself (Figure 4, right). This interpretation was supported by DFT calculations showing that the structure for the oxidized enzyme did indeed possess a persulfide. As shown in Figure 4 (left), the L_2_Mo^IV^(S‐SCys) configuration can be thought of as a resonance form of the canonical L_2_Mo^VI^S(S‐Cys) configuration, with neither fully capturing the true electronic character of the oxidized enzyme. The upshot, however, is that the reaction mechanism is fundamentally unchanged: it still involves hydride transfer from the formate to the same sulfur atom (alternately represented as the Mo^VI^═S or terminal persulfide sulfur), yielding the same L_2_Mo^IV^(SH)(S‐Cys) product (whose structure is not in question). The issue is whether the electron density added to the oxidized center has gone predominantly to reduce the metal or cleave the persulfide bond.

**FIGURE 4:**
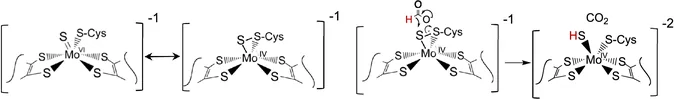
(Left) Alternate resonance forms for the oxidized molybdenum center. (Right) A mechanism for the oxidation of formate starting from the persulfide‐Mo(IV) form. In the lefthand resonance form, the cysteinate sulfur is formally negatively charged, and the terminal sulfido doubly so. In the righthand resonance form, the proximal sulfur of the cysteine persulfide is coordinated to the molybdenum via one of its lone pairs in a dative bond. Because both electrons are credited to the ligand in a formal valence count, this sulfur is neutral. The distal sulfur, on the other hand, is negatively charged as are those of the pyranopterin cofactors. The alternate resonance forms reflect the extent to which electron density is smeared over the molybdenum and two sulfurs. In the mechanism at right, the cysteinate sulfur and thiolate sulfur of product are formally negatively charged, and addition of hydride to the overall complex increases its overall charge from −1 to −2.

Summarizing the argument against the bicarbonate mechanism as proposed [12], the following can be said. First and foremost, no oxygen derived from solvent is observed to be incorporated into product CO_2_ under single‐turnover conditions (and after overnight preincubation of enzyme in ^18^O‐labeled water) [13], and it is impossible to imagine bicarbonate being generated from formate in a manner that does not involve a reaction with water. Second, the bicarbonate mechanism invokes displacement of cysteine from the molybdenum coordination sphere by hydroxide, with the dissociated cysteinate subsequently abstracting a proton from the hydroxide to initiate nucleophilic attack on formate. This being the case, lowering the pH should significantly retard the reaction as the cysteine spontaneously protonates from solvent. Instead, the rate constant for reduction of enzyme by formate does not decrease significantly, but increases by a modest two‐ to three‐fold as the pH is lowered from 9.5 to 6 [13]. Third, it can only be emphasized that other evidence cited in support of the bicarbonate mechanism [12] regarding formation of monothiocarbonate upon enzyme action on thioformate is problematic, relying as it does on the assignment of a feature at 183 ppm in the ^13^C NMR to monothiocarbonate (this on the basis of a 186 ppm feature of monothiocarbonate, generated by bubbling COS into 8 N KOH [20]). In the experiment described, however, this feature decays with a half‐life of ≈2 h, while the half‐life of monothiocarbonate in aqueous solution is known to be less than 100 ms at pH 9 [21]. Furthermore, this same work has shown that monothiocarbonate decays to CO_2_ and HS^−^ orders of magnitude faster than it dehydrates to COS and HO^−^ [21], yet it is claimed that a feature seen at 159 ppm in the ^13^C NMR is due to COS, which accumulates extensively by the end of the reaction. Given the preferred breakdown of monothiocarbonate to CO_2_ and HS^−^ and assuming that the 159 ppm feature in the ^13^C NMR experiment in fact corresponds to COS, it could only have been formed as the direct product of the oxidation of monothioformate by the enzyme in a reaction analogous to the oxidation of formate to CO_2_ and not by dehydration of monothiocarbonate. In sum, there are several serious questions concerning the interpretation of this NMR experiment using thioformate as substrate.

It must also be kept in mind that the evidence for dissociation of (seleno)cysteine in the course of catalysis by the metal‐dependent formate dehydrogenases that has been reported [9] is, in fact, quite weak. Indeed, no carboxamidomethylation of cysteine or enzyme inhibition was observed in the course of enzyme turnover with formate and NAD^+^ in the presence of iodoacetamide. Loss of activity and modification of the cysteine were only observed when nitrate was added to the reaction mix, which was assumed to interact with the molybdenum center in the same way as formate. A subset of the nitrate reductases in fact bear a striking structural homology to the formate dehydrogenases [22, 23, 24], but nitrate binds to the reduced molybdenum center of these nitrate reductases while formate binds to the oxidized molybdenum center of the formate dehydrogenases. As a result, nitrate is not expected to be a competitive inhibitor (with respect to formate) of the formate dehydrogenases. It is unfortunate that there was no further characterization of formate dehydrogenase inactivated by iodoacetamide in the presence of nitrate to ascertain whether the molybdenum center otherwise remained intact. It has recently been concluded that dissociation of the (seleno)cysteine is, in fact, the result of aerobic breakdown of the metal centers of these enzymes [25, 26].

Finally, it is to be noted that the bicarbonate mechanism has been inaccurately compared [12] to the established mechanism for the hydroxylation of xanthine by xanthine oxidase [27, 28] and referred to inappropriately as involving oxygen atom transfer as defined by Holm [29, 30, 31, 32]. The Holm definition of oxygen atom transfer involves straightforward transfer of an oxygen atom to or from a substrate lone pair of electrons. Molybdenum‐containing enzymes catalyzing such oxygen atom transfer reactions—including DMSO reductase, sulfite oxidase, and at least some of the nitrate reductases—do indeed catalyze such reactions consistent with the thermodynamic scale presented by Holm and Donahue [33]. The reaction catalyzed by xanthine oxidase, on the other hand, involves cleavage of a C─H bond and does not fall on the Holm/Donahue oxygen atom transfer scale at all. It is important not to conflate these two fundamentally different types of reactions, as Holm himself had earlier cautioned [34].

## Funding

This work was supported by the U.S. Department of Energy (Grant DE‐SC0010666).

## Conflicts of Interest

The authors declare no conflicts of interest.

## Data Availability

The data that support the findings of this study are available on request from the corresponding author. The data are not publicly available due to privacy or ethical restrictions.
